# Double-Layer Detection Model of Malicious PDF Documents Based on Entropy Method with Multiple Features

**DOI:** 10.3390/e25071099

**Published:** 2023-07-23

**Authors:** Enzhou Song, Tao Hu, Peng Yi, Wenbo Wang

**Affiliations:** Information Technology Institute, Information Engineering University, Zhengzhou 450001, China; feiyunyt@gmail.com (E.S.); yipengndsc@163.com (P.Y.); wbwangedu@outlook.com (W.W.)

**Keywords:** PDF document detection, multiple features, entropy method, AdaBoost-optimized random forest algorithm, robustness-optimized support vector machine algorithm

## Abstract

Traditional PDF document detection technology usually builds a rule or feature library for specific vulnerabilities and therefore is only fit for single detection targets and lacks anti-detection ability. To address these shortcomings, we build a double-layer detection model for malicious PDF documents based on an entropy method with multiple features. First, we address the single detection target problem with the fusion of 222 multiple features, including 130 basic features (such as objects, structure, content stream, metadata, etc.) and 82 dangerous features (such as suspicious and encoding function, etc.), which can effectively resist obfuscation and encryption. Second, we generate the best set of features (a total of 153) by creatively applying an entropy method based on RReliefF and MIC (EMBORAM) to PDF samples with 37 typical document vulnerabilities, which can effectively resist anti-detection methods, such as filling data and imitation attacks. Finally, we build a double-layer processing framework to detect samples efficiently through the AdaBoost-optimized random forest algorithm and the robustness-optimized support vector machine algorithm. Compared to the traditional static detection method, this model performs better for various evaluation criteria. The average time of document detection is 1.3 ms, while the accuracy rate reaches 95.9%.

## 1. Introduction

In recent years, the number of network attacks through malicious documents has increased dramatically. Such attacks are often accompanied by serious harm, such as phishing, organization monitoring, and denial of service attacks. In network attacks based on malicious documents, the PDF document type accounts for a large proportion. According to the statistics of F-Secure Security, in 2020, malicious document attacks related to Adobe Reader accounted for 60% of total document attacks. Attackers can use embedded scripts, remote links and other means to carry out attacks through the plentiful functions of Adobe related products. The detection of such concealed attacks is difficult [1].

Among PDF-related attacks, constructing document vulnerabilities by exploiting defects of Adobe software is extremely harmful [2,3,4,5]. Through exploiting the vulnerabilities of document readers or parsers, such attacks can cause various types of harm, including downloading malicious programs remotely, implementing backdoor implantation, and executing malicious code directly. In 2020, Adobe released a security update bulletin to disclose the vulnerability CVE-2020-24432, the principle of which is that Adobe Acrobat Reader lacks strictness while censoring input validation. Attackers can execute arbitrary code in the context of the current user and cause serious damage.

Since 2018, Adobe Acrobat Reader has released more than 30 security update bulletins and disclosed more than 200 CVE vulnerabilities. Among them, there are more than 50 document vulnerabilities which are destructive and widely disseminated. Table 1 presents the typical document vulnerability information disclosed by Adobe in recent years. It can be seen that document vulnerabilities are usually accompanied by harmful attacks, such as arbitrary code execution.

In recent years, researchers have presented various methods for detecting malicious documents. The detection types are mainly divided into two categories: static and dynamic detection methods. The static method usually determines the document’s nature through analyzing the content, basic attributes, basic structure, metadata, and other document features without running them [6,7,8,9], whereas the dynamic method usually achieves detection through analyzing the system calls, operation behavior, and other features in a virtual environment’s running process [10,11,12]. Traditional static and dynamic detection methods have advantages and limitations. Static detection does not need to execute actual samples and is thus relatively secure with high detection efficiency, fast speed, and low cost, but it ignores the malicious code extraction from documents [13]. Dynamic detection does not need to learn samples and can intuitively find the purpose of attack via running behavior, leading to a strong robustness. However, it faces such challenges as low efficiency, low speed, enormous cost, and sometimes threats from the anti-virtual machine and anti-sandbox technology [14,15,16].

Based on the above analysis, we propose a new double-layer detection model for malicious PDF documents based on an entropy method with multiple features. First, we integrate a total of 222 basic and dangerous features, such as PDF document objects, physical logical structure, content stream, metadata, JavaScript code, dangerous functions and bytes, etc. Second, we use an entropy method based on RReliefF and MIC to filter redundant features and select 153 features as the best feature set. Third, we train the double-layer model with the optimized random forest and the support vector machine classification model through the training set of 4121 malicious samples and 4721 benign samples. Finally, we design a comparative experiment and verify the reliability of the model through the test set of 1374 malicious samples and 1574 benign samples. The result shows our model has an accuracy rate of 95.9% and is better than the contrast model.

The main contributions of this paper are as follows:

(1) We propose a multi-feature fusion extraction method. It not only extracts basic features such as PDF document objects, physical structure, and content stream but also common dangerous features based on frequency induction, which solves the problem of a single detection target faced by conventional detection methods and effectively resists obfuscation and encryption.
(2) We present a feature-selection-filtering module: EMBORAM. Combined with the weights of features calculated by RReliefF and correlation of features and tags calculated by MIC, the best feature set generated by the entropy method can resist anti-detection methods such as data filling and imitation attacks.
(3) We construct a double-layer processing framework through the AdaBoost-optimized random forest and the robustness-optimized support vector machine classification model. The deficiencies of models in PDF document detection are improved and the detection effect is enhanced through optimization and combination.
(4) We collect a large amount of data with a comprehensive coverage of the training samples. Experiments verify that the detection accuracy rate is high at low time consumption. As the result shows, this model can efficiently detect malicious PDFs adopting common attack methods.

The rest of this paper is organized as follows. Section 2 introduces background and related work. Section 3 discusses in detail the double-layer detection model and the specific implementation of each module. Section 4 introduces the comparison experiments and analysis. Section 5 summarizes the work and gives an outlook.

## 2. Background and Related Work

This module mainly introduces the background of PDF and analyzes the realization and shortcomings of traditional methods.

### 2.1. PDF Background

**Figure 1:**
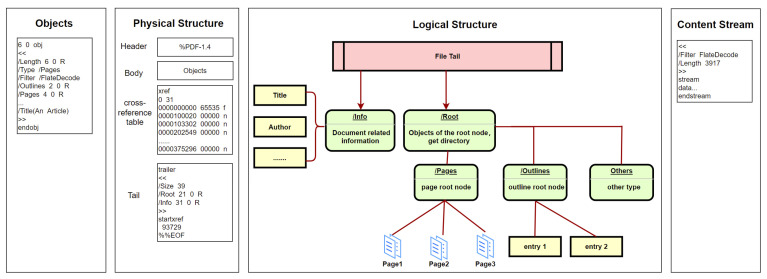
The basic structure of PDFs.

The newest format of PDF was published as ISO 32000-1:2020 [17]. According to the standard, the basic structure of PDF documents is mainly divided into four parts: objects, physical structure, logical structure, and content stream [18,19], as shown in Figure 1.

The details of each part are as follows. (a) Objects. As the main part of PDF documents, objects carry various content, such as text information, fonts, embedded pictures, embedded videos, hyperlinks, and bookmarks. But the basic structure of different objects is similar regardless of the content classification. As shown in Figure 1, the first line in objects is the identifier, which consists of two numbers. The first is the serial number of objects. The second is the generation number of objects and is used to indicate whether the object has been modified. (b) Physical structure. This is mainly composed of four parts: file header, file body, cross-reference table, and file tail. The file header with a simple and fixed format is used to indicate the PDF version. The file body composed of document objects is the core part of the PDF. The cross-reference table is used to index document objects. The file tail is mainly used to save the summary, location, and other related information of the cross-reference table. (c) Logical structure. In the actual parsing process, PDFs are not parsed through physical structure but through logical structure. Parsing begins with the root node indicated by the file tail. The node indicates the directory, which contains pages, outlines, and other types of information. Each type of information is also organized in a tree structure. (d) Content stream. This is a common form of objects in PDFs and plays a key role in storing data. Stream objects are composed of three parts. The first part is a dictionary, which mainly stores the length and encoding method. The second part is the keyword, which is unified in different stream objects. It usually starts with “stream” and ends with “endstream”. The third part is the data between keywords.

### 2.2. Static Detection Method

Currently, the common static detection methods are mainly divided into three categories. The first category tends to detect the content features of files, mainly to extract suspicious JavaScript code fragments, shellcode data fragments, and metadata content in PDF documents. According to Tzermias et al. [11], more than 90% of malicious PDF document attacks need to be implemented with JavaScript and other codes. The detection model proposed by Laskov et al. [20] extracts JavaScript and uses lexical tagging to build an OCSVM classification. However, such methods are insufficient for PDF documents that do not rely on JavaScript code. Some document vulnerabilities build attack chains with the help of the document format. The second category tends to detect the structural features of documents. It achieves detection mainly through extracting document structure and combining features such as metadata. Šrndić et al. [21] extracted vast features of basic structure for PDF but also had limitations in extracting malicious features. Cohen et al. [7] adopted the SFEM method to extract features from the document structure. Chandran et al. [22] scanned the structure of the PDFs through PeePDF and used the GRU model to employ classification. Srndic et al. [23] processed metadata in a similar way to structural paths and then substituted the data into classification models to achieve detection. But such methods have limited abstraction of features and are not comprehensive enough to detect content features. The static methods above are not comprehensive in feature extraction, resulting in insufficient detection for various attack methods. The third category tends to build the feature library and use multiple features. Wen Weiping et al. [24] designed a feature library for document vulnerabilities. The malicious document can be identified when it matches the relevant features of the feature library. However, such methods only apply to malicious PDF documents with disclosed vulnerabilities and have no detection effect on 0-day vulnerabilities. Falah et al. [25] used feature engineering to evaluate multiple features and detect malicious PDFs, but they ignored malicious features in JavaScript code, and the feature evaluation method deserves improvement.

### 2.3. Dynamic Detection Method

Dynamic detection methods mainly focus on JavaScript code and shellcode data fragments embedded in the document. The MDScan method [14] mainly executes the extracted JavaScript code. It extracts the relevant operation performance of memory as a sequence and performs subsequent detection. But these similar matching methods have limitation in detecting new type of attacks. Iwamoto et al. [26] used the simulation method to execute the document shellcode. It is mainly based on the entropy of the byte sequence, which can solve the problem of difficult vulnerability triggering to some extent. However, this detection is insufficient for some malicious codes that can be only triggered in a specific situation. Xu et al. [27] proposed opening the PDF document with the same reader in the heterogeneous operating system and identified malicious documents through the similarity performance of system calls and process tracking. However, such methods have an excessive overhead and low detection efficiency. Liu et al. [28] executed JavaScript code in PDF documents through their own built-in execution environment and monitored common malicious behaviors. But such methods can only detect traditional and common attack methods. It is insufficient to detect the document using new anti-detection method. In summary, the dynamic detection method is expensive and consumes large amounts of resources and memory space. It is not suitable for situations with large numbers of samples, short time requirements, and low resource requirements [29].

## 3. Double-Layer Detection Model Based on Entropy Method with Multiple Features

In order to make up for the shortcomings of traditional detection methods, this paper strives to improve from the following perspectives: (a) comprehensive detection of malicious PDFs, which can achieve effective detection of a variety of vulnerability attacks; (b) effective defense against common anti-detection methods, such as data stuffing, mimicry attacks, etc.; (c) fast detection, low cost, and low consumption. Therefore, we design a double-layer detection model based on an entropy method with multiple features. This section focuses on the specific introduction of the model, including model outline, model framework, theoretical support, and specific implementation methods.

### 3.1. Overview of the Model

**Figure 2:**
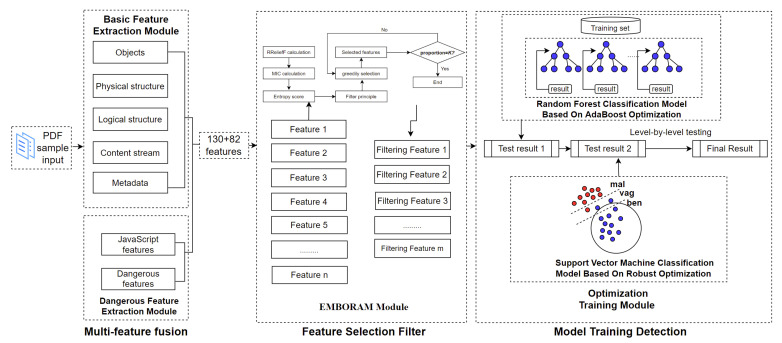
Model framework.

Based on the analysis of benign and malicious samples, it can be concluded that there are some differences between two types of samples in terms of basic and dangerous features. Therefore, we can effectively detect malicious documents through constructing the training model via supervised learning. The model framework of this paper is shown in Figure 2.

As the figure shows, we first extract and fuse the basic and dangerous features of the document. Then, we select and filter features through EMBORAM. Finally, we train the feature set in the optimized classification model. The detection model is mainly divided into four modules: the basic feature extraction module, dangerous feature extraction module, EMBORAM module, and optimization training module. The first two modules implement extraction and fusion of multiple features, the third module implements selection and filtering of features, and the fourth module implements optimization, training, and detection.

### 3.2. Basic Feature Extraction Module

Basic features of PDF are mainly divided into four parts: objects, physical structure, logical structure, and content stream. To extract PDF document features more comprehensively, this module also extracts metadata of PDFs.

The basic feature extraction work references four mainstream PDF document analysis tools, including PeePDF [30], PDFParser [31], PDFTear [32], and PDFRate [33]. The above tools can extract PDF features and analyze PDF maliciousness but have certain limitations. Therefore, this paper combines four mainstream tools with designed regular expressions to achieve a comprehensive extraction and fusion of PDF basic features.

The comparison between this paper and four mainstream analysis tools is shown in Table 2. As the table shows, we can ensure the comprehensiveness of the feature set and improve the detection accuracy.

First, we use the above tools to extract features from PDF. Then, we use regular expressions to match different feature symbols. After that, we can extract the specific feature content and its corresponding information, such as quantity, location, etc. The symbols matched by regular expressions in this paper are shown in Appendix A.

Therefore, with the help of four mainstream PDF document analysis tools and the designed regular expressions, this module extracts a total of 130 basic features of PDF documents. Detailed information on basic features extracted in this paper is shown in Appendix B.

### 3.3. Dangerous Feature Extraction Module

Dangerous feature extraction is mainly for PDF documents with JavaScript attacks. After analyzing a number of document vulnerabilities, we found that malicious PDF generally achieve attack by PDF reader vulnerabilities. Among them, buffer overflow attacks account for the majority. In order to achieve command execution after the buffer overflow, the attack usually needs to work with JavaScript. Therefore, the dangerous feature extraction mainly focuses on JavaScript features.

Fernandez et al. [34] collected and classify samples of malicious code of web pages, which are divided into different categories. Each category contains a type of malicious JavaScript code and its variations. The code in malicious PDF is similar to that in malicious web pages. But JavaScript in PDFs has some characteristics of its own. Therefore, we need to add some malicious features for PDFs to create a comprehensive extraction. We take the CVE-2020-24432 sample as an example. The vulnerability achieves arbitrary command execution through the input validation defect of Adobe.

The attack is exploited through overflow vulnerability. It hijacks the return address to the filling data built by JavaScript and finally executes malicious JavaScript code. We can find that the malicious code often creates obfuscation, encoding, and decoding operations. By highly obfuscating JavaScript code, it can effectively counteract the detection of malicious JavaScript in PDFs. De-obfuscating such code is also difficult without running it. However, we can extract dangerous features of JavaScript as training data and achieve detection through multidimensional feature classification rather than JavaScript code analysis.

Therefore, we must summarize common malicious JavaScript operations and use them as our dangerous feature set. We analyze 10 types of typical malicious PDF files that attack through JavaScript code, as shown in Table 3.

We use the basic feature extraction method to extract the JavaScript objects of the above malicious PDF files. To extract and summarize the dangerous features, we need to perform the corresponding word separation operation in the JavaScript code and count the frequency. We use the special character separation method. The special characters for separation are: “,”; “(”; “)”; “%”; “;”; “+”; “/”; “‘”; “=”; “<”; “>”; “ ”. We divide characters into four categories according to the feature type: (1) Basic attribute features. (2) Redirection features. (3) Suspicious keyword features. (4) Confusion features.

**Figure 3:**
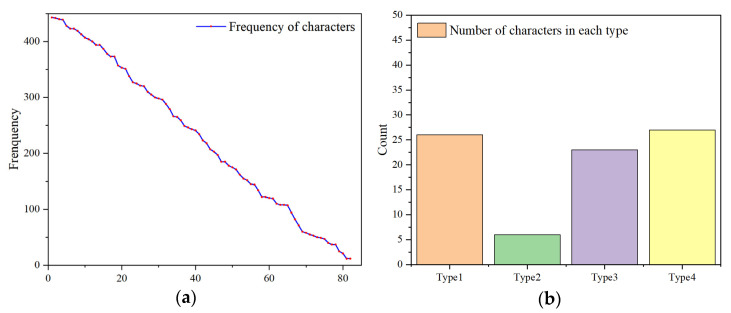
(**a**) Frequency of characters in malicious PDFs; (**b**) number of characters in each type.

For the above PDF files, we analyzed word frequency, and the final results are shown in Figure 3.

As shown in the figure, the number of characters extracted in JavaScript is 82, and the number of characters in each type are 26, 6, 23, and 27, respectively. In addition, we add some macro features, e.g., the proportion of specific characters. The features summarized in this paper are shown in Appendix C.

### 3.4. EMBORAM Module

**Figure 4:**
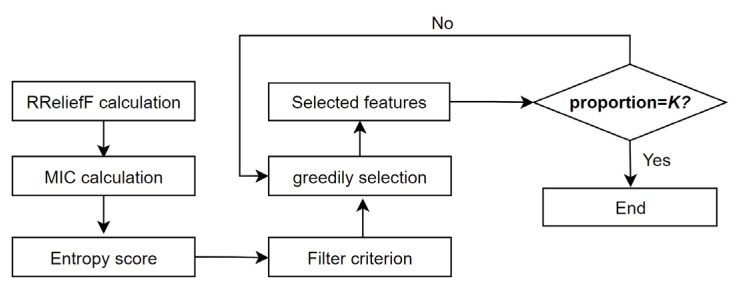
Frame of EMBORAM module.

In the previous section, we implemented multi-feature fusion extraction and extracted a total of 222 relevant features from PDF documents. But the number of features is not necessarily proportional to the detection accuracy. Some of them are redundant features, which have a negative impact on the detection accuracy. Therefore, we need to perform feature selection and filtering to eliminate redundant features and improve the detection accuracy of the model. In this module, we creatively raise the entropy method based on RReliefF and MIC (EMBORAM). According to the weights of features calculated using RReliefF and correlation of features and tags calculated using MIC, we use an entropy method to score each feature and set a filter criterion to greedily select the best feature set. Because the attacker does not know the details of the set, the filler or imitation data constructed by the attacker have a certain probability of being filtered by this module. Therefore, we can not only improve the accuracy of detection but also effectively counter attacker’s anti-detection methods. The frame of the module is shown in Figure 4.

We set the initial number of PDF features as N(N=222). The RReliefF algorithm can discover the strong dependencies between attributes and has strong robustness. It also can be used for classification tasks. For the feature $A_{i}$, it chooses g (usually g=5) nearest PDF samples and calculates the importance of $A_{i}$ through the distance of the samples. The weight of each feature calculated by RReliefF is $r,r={\left[W\left[A_{1}\right],W\left[A_{2}\right],\ldots ,W\left[A_{N}\right]\right]}^{T}$. MIC can calculate the correlation of features and tags through maximum mutual information values. We note the tag of the PDF dataset as C(C∈{ben,mal}). For each feature $A_{i}$ and tag C, we use MIC to obtain the final result $q={\left[p\left(A_{1}:C\right),p\left(A_{2}:C\right),\ldots ,p\left(A_{N}:C\right)\right]}^{T}$.

The entropy method is excellent and objective in this assignment field. It is based on the degree of variation of indicators. First, we normalize r and q as calculated by RReliefF and MIC, as in the following equation.

(1) $$r_{new}=\frac{r_{i}-\min\left(r\right)}{\max\left(r\right)-\min\left(r\right)},\;q_{new}=\frac{q_{i}-\min\left(q\right)}{\max\left(q\right)-\min\left(q\right)}$$

Then, we calculate the information entropy of r and q according to the normalization result, as in the following equation.

(2) $$E_{r}=-\frac{1}{lnN}\sum_{i=1}^{N}P_{r_{i}}lnP_{r_{i}},\;E_{q}=-\frac{1}{lnN}\sum_{i=1}^{N}P_{q_{i}}lnP_{q_{i}},\;\;P_{r_{i}}=\frac{r_{i}}{\sum_{i=1}^{N}r_{i}},\;P_{q_{i}}=\frac{q_{i}}{\sum_{i=1}^{N}q_{i}}$$

Therefore, the weights of r and q can be separately calculated, as in the following equation.

(3) $$\eta =1-\frac{1-E_{r}}{2-{(E}_{r}+E_{q})}\;,\;\theta =1-\frac{1-E_{q}}{2-(E_{r}+E_{q})}$$

Then, we build a filter criterion as in Equation (4), which represents the weighted average for the relevance of the new feature set to the category. S represents the selected feature set which contains features s. f represents the feature to be selected. η and θ balance the importance of RReliefF and MIC.

(4) $$\eta *\frac{\sum r\left(s\right)+r\left(f\right)}{\left|S\right|+1}+\theta *\frac{\sum q\left(s\right)+q\left(f\right)}{\left|S\right|+1},\;s\epsilon S$$

Finally, a forward search strategy is adopted to greedily select the features that maximize the criterion until the number of features reaches the selection proportion threshold K (after the final experiment. The best result is achieved when K=0.69 while the number of feature set is 153). We use the finalized feature set as our selected features for model training and thus minimize the interference of redundant data.

### 3.5. Optimization Training Module

In this paper, we use the random forest and the support vector machine classification models. Both of them are suitable for processing high-dimensional data, and thus—in the case of PDF documents with a large number of features—the model can be trained with less computational overhead and greater robustness. But both classification models have certain drawbacks for PDF document detection. Therefore, we implement some optimization and design a double-layer detection model. In this way, we can make up the shortcomings of both models, which are unstable and susceptible to noise, to some extent. 

#### 3.5.1. AdaBoost-Optimized Random Forest Classification Model

The original random forest classification model has some limitations. The classifier will be over-fitted if there is noise in the features. Although we use EMBORAM to select the best features in the early section, there may still be noise in the feature set. Therefore, we create AdaBoost-optimized random forest classification to improve the overall training and classification effect.

**Figure 5:**
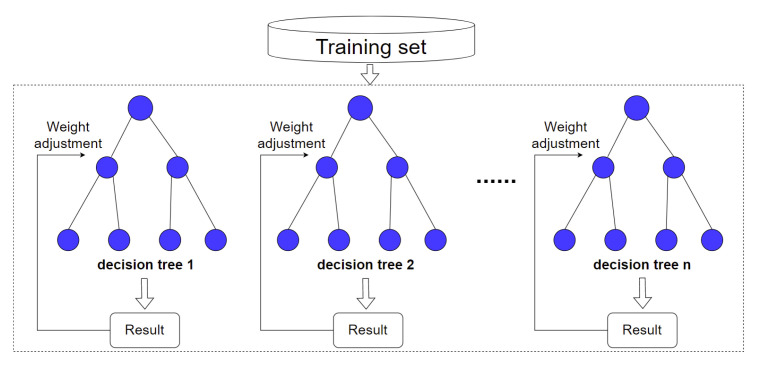
AdaBoost-optimized random forest classification model.

AdaBoost is essentially an adaptive boosting algorithm. It can adjust the weights of each classification according to the accuracy. Random forest collects samples randomly to form subsets. Each subset builds a decision tree after training. Finally, it uses decision trees to build the random forest and conducts classification by equally voting. To improve the effect, we use the AdaBoost algorithm to adjust voting weights of decision trees in random forest. Decision trees with stronger classification ability have higher voting weights. We obtain the final results through the maximum voting principle. The optimized method is as shown in Figure 5.

We note the extracted features for each PDF sample as F and note the number of decision trees as T. The dataset is $\left\{\left(x_{1},y_{1}\right),\left(x_{2},y_{2}\right),\ldots ,\left(x_{n},y_{n}\right)\right\}$, where $x_{i}\in F$, $y_{i}\in Y=\{ben,mal\}$. We note the initial classifier as $H,\;H=\{h_{1},h_{2}\ldots h_{T}\}$. The optimization steps are as follows.

(1) We use the dataset and features F to build the initial decision trees $h_{t}\left(x\right)$ in the random forest.
(2) The classification weight of the decision trees for each category is calculated based on the classification ability. We initialize training sample weights, which means the weight of decision tree t for sample i, as in the following equation.

(5) $$\;\;w_{i}^{t}=\frac{1}{n},\;\;\;i=1,2,\ldots ,N$$

For the decision trees t=1:T, we loop from (a) to (c).

(a) We calculate the error rate $\varepsilon_{t}^{C}$ of the decision tree $h_{t}(x)$ to the category C, as in the following equation.
  (6) $$\varepsilon_{t}^{C}=\sum_{i=1}^{N}w_{i}^{t}*sign(h_{t}(x_{i})\neq C)$$
(b) If $\varepsilon_{t}^{C}>1/2$, the decision tree will be dropped and this cycle will be finished. Because AdaBoost is designed to binary classification algorithm, which requires the error rate to be less than 1/2 (random guessing probability).
(c) If $\varepsilon_{t}^{C}<1/2$, we calculate the weight of the decision tree $h_{t}(x)$ for the category C, as in the following equation.
  (7) $$\alpha_{t}^{C}=\frac{1}{2}\log\left(\frac{1-\varepsilon_{t}^{C}}{\varepsilon_{t}^{C}}\right)$$
(d) We can update the weight of decision tree $h_{i}$ in the random forest, as in the following equation.
  (8) $$w_{i}^{t}=\frac{w_{i}^{t}}{Z_{i}}\exp\left(\alpha_{t}^{C}*sign(h_{t}(x_{i})\neq C)\right)$$

$Z_{i}$ is the normalization factor, as in the following equation.

(9) $$Z_{i}=\sum_{i=1}^{n}w_{i}^{t}\exp\left(\alpha_{t}^{C}*sign(h_{t}(x_{i})\neq C)\right)$$

The $\alpha_{t}^{C}$ is the weight of decision trees for the category C.

(3) We sequentially set C=ben,mal and repeat step (2). The final voting weights of the decision trees for two categories can be obtained.

#### 3.5.2. Robust Optimized Support Vector Machine Classification Model

The support vector machine has the advantage of high-dimensional feature-handling and meets the need for PDF document classification. However, it is more suitable for situations with small sample sizes. For situations such as malicious PDF detection that require a large number of samples for training, there is a problem of unstable classification. Therefore, we perform robustness optimization of the support vector machine. Christmann, A. et al. [35] proposed an HL-SVM model. They imported the regularization parameter and constrained some unstable samples in the training data.

We note the extracted features for each PDF sample as F and note the training set of all PDF documents as T, $T=\{\left(x_{1},y_{1}\right),\left(x_{2},y_{2}\right),\ldots ,\left(x_{n},y_{n}\right)\}$, where $x_{i}\in F$, $y_{i}\in Y=\{ben,mal\}$. The optimization equation for the HL-SVM model is as follows.

(10) $$\min\left(a,b\right)\left\{\frac{\lambda }{2}{∥a∥}_{2}^{2}+\sum_{i=1}^{n}{\left(1-y_{i}\left(a^{T}x_{i}+b\right)\right)}_{+}\right\}\;a\in R,\;b\in R$$

**Figure 6:**
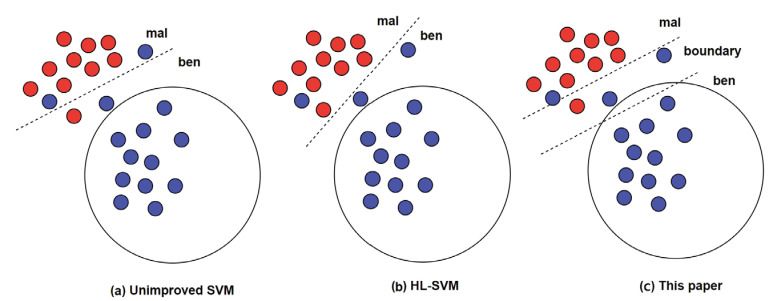
Robustness-optimized support vector machine classification model.

Among them, $a^{T}x_{i}+b=0$ is the classification hyperplane of the support vector machine; λ>0 is the regularization parameter; the function ${\left[\;\right]}_{+}=max[0,1-y_{i}\left(a^{T}x_{i}+b\right)]$ constrains the samples which affect the stability and accuracy of the hyperplane. But such a method will cause great changes in the hyperplane and reduce the accuracy for future samples, as shown in Figure 6b.

Therefore, we improve the optimization equation, as in the following equation.

(11) $$\min\left(a,b,\theta \right)\left\{\frac{\lambda }{2}{∥a∥}_{2}^{2}+\sum_{i=1}^{n}\theta_{i}{\left(1-y_{i}\left(a^{T}x_{i}+b\right)\right)}_{+}\right\}\;a\in R,\;b\in R,\theta \in {\left(\theta_{1},\theta_{n}\right)}^{T}\in {\left[0,1\right]}^{n},\sum_{i=1}^{n}\theta_{i}≧n(1-\theta )$$

We set a boundary range by moving the hyperplane. $\theta_{i}$ is used to judge whether the sample is in the boundary set. If the sample meets the boundary range, then $\theta_{i}=0$, and it is rejected from participating in the learning of the support vector machine model, as shown in Figure 6c.

As can be seen in the figure, the improved support vector machine incorporates unstable samples into the learning range, resulting in limitation in classification stability. The HL-SVM model causes great changes in the hyperplane. In contrast, the robustness-optimized support vector machine rejects the boundary samples from participating in model learning so that the overall stability of the model is improved. Therefore, the choice of the boundary sample selection will have an impact on model training. We note the width of boundary range as B. After the final experiment, the best result is achieved when B=0.13, while the number of boundary set is 271.

#### 3.5.3. Double-Layer Processing Framework

We use the AdaBoost-optimized random forest model and the robustness-optimized support vector machine model as classifiers. To obtain the final detection model, we need to combine the two classifiers. We build a double-layer processing framework. We first use the random forest model and then use the support vector machine model for detection. We note the result of random forest model detection as ${result}_{1}$, the result of support vector machine model detection as ${result}_{2}$, and the sample classification set as {ben,mal}. The final detection result is as follows.

(12) $$result={result}_{1}⋃{result}_{2}\left(ben=0,mal=1\right)$$

When both detection models detect the sample as benign, the file detection result is benign. Through double-layer processing, the problem of false negatives can be solved best and the overall detection ability of the model can be improved.

## 4. Experiment and Test

### 4.1. Dataset

This section focuses on the sample sources used for the experiments and tests. The benign samples were collected mainly by Google [36], Yahoo Search Engine [37], and the Github react-pdf project [38]. To enhance the robustness of the experiments, we collected benign samples extensively, including various types of PDF samples with advanced features. All benign samples were scanned and checked using VirusTotal. The malicious samples were downloaded through VirusTotal. The set contains samples of malicious PDF documents from more than 100 exploits during the last 10 years. We selected a total of 5495 malicious PDF samples with 37 typical vulnerabilities from the set. Among the malicious samples, there are 1074 samples with high obfuscation and encryption (malicious-high samples). All the samples were detected as malicious through VirusTotal and sandbox. The basic attributes of the samples are shown in Table 4.

According to the statistics of the basic attributes, it can be seen that more than 60% of the malicious samples are smaller than 100 KB. But most of the benign PDFs crawled through search engines are larger than 100 KB in size. Therefore, we used the react-pdf project to create some PDF files smaller than 100 KB in size. In this way, the robustness of the model can be improved because the features of these samples fit the malicious documents.

### 4.2. Environment

The experiment is conducted using an NVIDIA RTX3090 GPU, connected by an Intel (R) Core (TM) i7-1165G7 @ 2.80 GHz. The software is Python3.7.1. In terms of settings for random forest model, the max_features is 0.7, n_estimators is 70, and max_depth is 8. In terms of settings for the support vector machine, the kernel function is rbf. In terms of dataset, we select 75% of benign and malicious PDF samples as the training set (4721 benign samples and 4121 malicious samples) and 25% as the test set (1574 benign samples and 1374 malicious samples) via the random sampling method.

### 4.3. Procedure

We first investigate the feature selection proportion threshold K and the boundary sample selection threshold B to obtain the best threshold value. Then, we substitute the value into the subsequent comparison experiments. After the experimental process, the accuracy and performance of the model are evaluated based on the records.

### 4.4. The Impact of Feature Selection Proportion Threshold K

**Figure 7:**
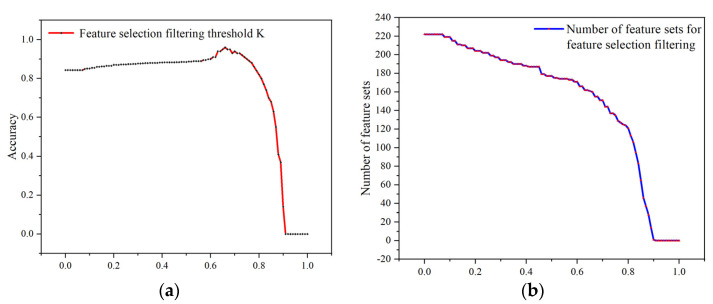
(**a**) Effect of the threshold K on accuracy; (**b**) effect of threshold K on the number of feature sets.

In the EMBORAM module, we utilize an entropy method based on RReliefF and MIC. In the final stage of filtering, we greedily select the features which maximize the filter criterion until the number reaches the setting proportion. The setting of the proportion threshold K affects the number of features in the final feature set. Therefore, the threshold K also has some influence on the accuracy of the model. In our experiments, we conducted traversal experiments on the threshold K. The impact effect is shown in Figure 7.

According to the figure, we know that when the threshold K is set too low, the number of the final feature set is high. Redundant features will participate in the training, which will have a negative impact on the model accuracy. In the interval of 0.67≤K≤0.77, the model accuracy has a certain volatility. When K>0.77, the number of the feature set is small. As a result, the number of the feature set is too low, leading to a negative impact on the model. When K=0.69 and the number of feature sets is 153, the model accuracy reaches the highest. At this time, we can filter redundant features and retain the best features to achieve optimal learning and training effect for the model.

### 4.5. Filtered Features

In the EMBORAM module, we filter the best dataset with 153 features by entropy method. The final number of features, filtered number of features, and filtered proportion in each category is shown in Table 5.

According to the result, the features in the objects category of basic features and the basic attribute category of dangerous features are mostly filtered. This reflects that differences in some objects of PDF samples are tiny and that the metric built using RreliefF and MIC is low, which finally leads to a lower score via the entropy method. The minimum filtered proportion is metadata features, which have the most obvious differences in PDF samples and are commonly used in relevant research of malicious PDF detection. Each category in our paper has its own selected features after being filtered, which shows the features extracted in the model are multiple and comprehensive.

### 4.6. The Impact of the Boundary Sample Selection Threshold B

**Figure 8:**
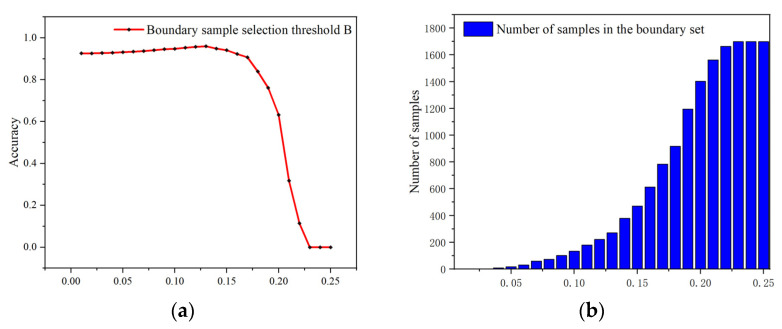
(**a**) Effect of threshold B on accuracy; (**b**) effect of threshold B on the number of samples in the boundary set.

In the training phase of the robustness-optimized support vector machine model, we define the boundary sample set. The robustness of the model is improved because the unstable samples that fall into the boundary sample set do not participate in the model learning of the support vector machine. Therefore, the method of dividing the boundary sample set affects the model training to a certain extent. According to the description in the previous section, we divide the boundary sample set by translating the classification hyperplane up and down, so the boundary sample set threshold B has some influence on model learning. We conduct experiments on threshold B, and the effect is shown in Figure 8.

According to the figure, we know that when the boundary sample selection threshold is set too low, the number of samples in the boundary set is small. Almost all samples can participate in the support vector machine model learning, resulting in the negative effect on the stability of the model to some extent. When the threshold value grows, the number of samples in the boundary set is moderate. At this time, the vast majority of boundary samples can be removed from the training set, leading to the positive effect on the stability of the model. When the threshold value is set too high, most of the normal samples are divided into the boundary set and the actual number of training samples is small, causing a significant decrease in the accuracy rate. When B=0.13 and the number of samples in the boundary set is 271, the model accuracy reaches its highest.

### 4.7. Evaluation Indicators

In this section, we design comparative experiments with other static detection models and conduct analysis. We mainly use *Precision*, *Recall*, and F1 values as evaluation criteria. The Precision is the probability that the alert is actually malicious among all the samples. The Recall is the probability that the alert is actually malicious among all the malicious samples. The F1 value is the equilibrium value that allows both precision and recall to reach the highest value, which is called Fscore.

(13) $$Precision=\frac{TP}{TP+FP}$$

(14) $$Recall=\frac{TP}{TP+FN}$$

(15) $$Fscore=\frac{2TP}{2TP+FP+FN}$$

where TP represents the number of samples which are malicious and successfully identified by the model. FP represents the number of samples which are benign but wrongly identified by the model, i.e., the number of false positive samples. FN represents the number of samples which are malicious but not identified by the model, i.e., the number of missed samples.

### 4.8. Contrast Model

The comparison models in this section are mainly divided into four categories, and each category contains two types of detection models: (1) traditional random forest and support vector machine detection models without EMBORAM, denoted as Tra_RF,Tra_SVM; (2) traditional random forest and support vector machine detection models with EMBORAM, denoted as Imp_RF,Imp_SVM; (3) optimized random forest and support vector machine detection models without EMBORAM, denoted as Tra_AdaRF,Tra_RobSVM. (4) related research in PDF detection [23,24], denoted as Smutz,Srndic. The model in this paper is denoted as Paper.

### 4.9. Evaluation of Competency

**Figure 9:**
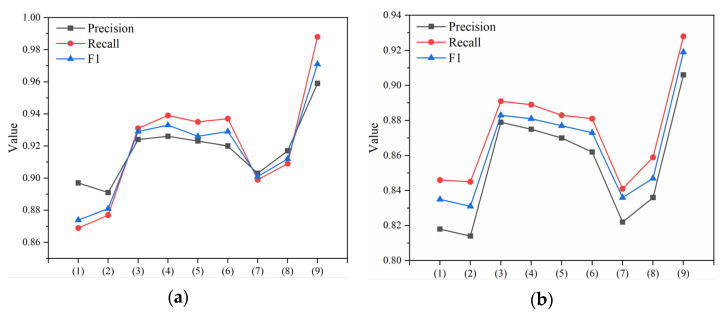
(**a**) Comparison of detection results in malicious samples; (**b**) comparison of detection results in malicious-high samples.

The evaluation is divided into two main parts. The first part is for all malicious samples. The Fscore of the paper reaches its highest when the threshold is 0.97. The accuracy of the model reaches 0.959, and the recall reaches 0.988. The second part is for highly obfuscation and encryption samples. The Fscore of the paper reaches its highest when the threshold is 0.96. The accuracy of the model reaches 0.906, and the recall reaches 0.928. In two parts, indicators are better than the comparison models, indicating that filtering the selection of features and optimization of the classification models can effectively improve the model’s detection effect. The *Precision*, *Recall*, and F1 results of the nine detection models are shown in Table 6 and Figure 9.

Based on the detection results for all malicious samples, we can find that the Tra_RF,Tra_SVM traditional static detection models based on the feature set extracted by this paper also achieve high accuracy and recall rate. This proves that the extracted features in this paper are comprehensive. The detection effect can be better achieved by extracting both the basic and dangerous features of the document.

The accuracy of Imp_RF,Imp_SVM detection models is increased considerably over the traditional detection model. This proves that the selection and filtering of the features using EMBORAM has an obvious positive effect on the classifier training. The reason for this situation is that some features in the vast majority of PDF documents may only exhibit a slight difference. Such features will have a certain degree of impact and interference on the classifier learning, resulting in a negative effect.

The Tra_AdaRF,Tra_RobSVM detection models show a large improvement over traditional common detection model. This proves that optimization of the classifier will improve the detection effect of the model. It should be noted that the Imp_RF,Imp_SVM detection models have a smaller improvement in accuracy rate than the Tra_AdaRF,Tra_RobSVM detection models, indicating that the selection of the feature set has a better improvement effect compared to the optimization of the classifier. This also shows that the capabilities of the two classification models do not show a large difference.

The model in this paper also has an improvement over related research, such as Smutz,Srndic. This reflects that features extracted in this paper are more comprehensive because we realize the fusion of multiple features, especially for the dangerous features in JavaScript.

Based on the detection results for malicious-high samples, we can find that both *Precision* and *Recall* are reduced. But the comparison models Smutz and Srndic reduce the most, which indicates multiple features extracted and filtered by the paper can effectively resist obfuscation and encryption.

**Figure 10:**
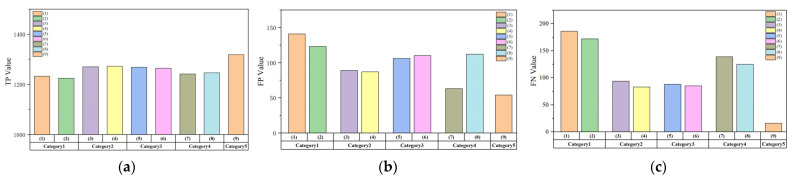
(**a**) Comparison of TP values; (**b**) comparison of FP values; (**c**) comparison of FN values.

In order to explore the performance differences between our model and comparison models more intuitively, we count the TP, FP, and FN values of several models for all malicious samples and obtain the results shown in Figure 10.

According to the figure, this model has better performance than other compared models in terms of TP, FP, and FN values. We can find that the number of false positives in Category 3 is significantly higher than in Category 2 because it does not select extracted features. Some common features in benign and malicious samples can confuse the model and affect the model training. It is worth noting that if a longitudinal comparison is made between the FN and FP values, it can be found that the number of false positives is higher than the number of missed positives in this model due to the use of a double-layer processing framework. The sample is identified as malicious as long as one of the classification models alarms, so the model can maintain a small number of missed positives better.

### 4.10. Time Overhead

The time overhead mainly contains training time and detection time. In the training phase, we processed a total of 8842 samples. We extracted 1,962,924 features and obtained 1,352,826 features after selection and filtering. The training time mainly contains four stages. The time spent in each training stage is shown in Table 7.

The detection time mainly contains three stages. In this phase, we processed a total of 2948 samples. We extracted 451,044 features and substituted them into the training model for detection. The average time spent is 1.3 ms, and the time spent in each detection stage is shown in Table 8.

For the detection time, the basic feature extraction time is the longest (0.76 ms). This is because the module extracts a large number of features and needs to parse each structure of the PDF document. However, the average detection time of the model is short and the overhead is low, which proves that the model is efficient.

## 5. Conclusions

In this paper, we design an efficient detection model for malicious PDFs which does not rely on feature libraries but on an entropy method with multiple features. The model is divided into four modules. In two feature extraction modules, we realize multi-feature fusion and extract a total of 222 basic and dangerous features of PDF documents. Through comprehensive feature set extraction, we can effectively resist obfuscation and encryption. In the EMBORAM module, we use entropy method based on RReliefF and MIC to select and filter the feature set according to the calculated criterion. We summarize the best set of 153 features, which can better resist data padding, imitation attacks, and other anti-detection methods. In the optimization training module, we build a double-layer processing framework through the AdaBoost-optimized random forest and the robustness-optimized support vector machine. Through the framework, we can improve the performance of two classification models in PDF document detection.

We process a total of 6295 benign samples and 5495 malicious samples. The experimental result shows that the EMBORAM and classifier optimization have significantly improved the model’s detection performance. The model can detect the common vulnerability attacks comprehensively and can effectively resist anti-detection methods such as data stuffing and imitation attacks. Furthermore, compared to the traditional static detection model, it has higher accuracy.

In future work, we will try to use more types of classification model and combine multiple classification models hierarchically to achieve better detection results.

## Figures and Tables

**Table 1:** Adobe typical document vulnerability information.

Number	Vulnerability	Dangerous Effects
CVE-2022-27787	Buffer overflow	Arbitrary code execution
CVE-2021-44709	Buffer overflow	Arbitrary code execution
CVE-2021-28564	Buffer overflow	Arbitrary code execution
CVE-2021-21017	Buffer overflow	Arbitrary code execution
CVE-2021-21045	Improper access control	Privilege escalation attack
CVE-2020-9704	Buffer overflow	Arbitrary code execution
CVE-2019-8249	Logical flaw	Arbitrary code execution
CVE-2019-8066	Buffer overflow	Arbitrary code execution
CVE-2018-19716	Buffer overflow	Arbitrary code execution

**Table 2:** Comparison of PDF basic feature extraction tool.

Tool	Features Included	Features Not Included
PeePDF	Content stream	Objects, physical structure, logical structure, metadata
PDFParser	Objects, metadata	Physical structure, logical structure, content stream
PDFTear	Content stream	Objects, physical structure, logical structure, metadata
PDFRate	Content stream, metadata	Objects, physical structure, logical structure
This paper	Objects, physical structure, logical structure, content stream, metadata	

**Table 3:** JavaScript attack vulnerability information.

Number	Vulnerability Causes	Hazard Impact
CVE-2022-34230	UAF attack	Arbitrary code execution
CVE-2022-27793	Command Injection	Arbitrary code execution
CVE-2022-27791	Buffer overflow	Arbitrary code execution
CVE-2021-44711	Buffer overflow	Arbitrary code execution
CVE-2021-44703	Buffer overflow	Arbitrary code execution
CVE-2021-39863	Buffer overflow	Arbitrary code execution
CVE-2019-8014	Buffer overflow	Arbitrary code execution
CVE-2017-16398	UAF attack	Arbitrary code execution
CVE-2011-0618	Buffer overflow	Arbitrary code execution
CVE-2010-2883	Buffer overflow	Arbitrary code execution

**Table 4:** Basic properties of samples.

Type	Quantity < 100 KB	Quantity 100–1000 KB	Quantity > 1000 KB	Sum
Benign	3918	1581	796	6295
Malicious	3637	1125	733	5495
Malicious-high	175	574	325	1074

**Table 5:** Filtered features.

Feature Attribute	Feature Category	Selected Number	Filter Number	Filtered Proportion
Basic Features	Objects	23	23	0.50
	Physical structure	14	5	0.26
	Logical structure	20	6	0.23
	Content stream	13	3	0.19
	Metadata feature	21	2	0.09
Dangerous Features	Basic attribute	20	23	0.53
	Redirection	5	1	0.17
	Suspicious keyword	18	5	0.22
	Confusion	19	8	0.30

**Table 6:** Comparison of model detection results.

Model Category	Number	Model Name	Malicious Samples			Malicious-High Samples		
			Precision	Recall	Fscore	Precision	Recall	Fscore
Category 1	(1)	Tra_RF	0.897	0.869	0.874	0.818	0.846	0.835
	(2)	Tra_SVM	0.891	0.877	0.881	0.814	0.845	0.831
Category 2	(3)	Imp_RF	0.924	0.931	0.929	0.879	0.891	0.883
	(4)	Imp_SVM	0.926	0.939	0.933	0.875	0.889	0.881
Category 3	(5)	Tra_AdaRF	0.923	0.935	0.926	0.870	0.883	0.877
	(6)	Tra_RobSVM	0.920	0.937	0.929	0.862	0.881	0.873
Category4	(7)	Smutz	0.903	0.899	0.901	0.822	0.841	0.836
	(8)	Srndic	0.917	0.909	0.912	0.836	0.859	0.847
Category 5	(9)	Paper	0.959	0.988	0.971	0.906	0.928	0.919

**Table 7:** Training phase time overhead.

Training Phase	Time Consumption (s)
Basic feature extraction	1973
Dangerous feature extraction	517
EMBORAM	1.82
Model optimization training	3.79

**Table 8:** Test phase time overhead.

Training Phase	Time Consumption (ms)
Basic feature extraction	0.76
Dangerous feature extraction	0.21
Model Detection	0.33

## Data Availability

Data can be obtained by contacting the author (feiyunyt@gmail.com, 16 May 2023).

## Appendix A

**Table A1:** Symbols Matched by Regular Expressions.

Feature Type	Symbols
Objects	obj, endobj, /Type, /FirstChar, /LastChar, /Widths, /BaseFont, /Subtype, /Ascent, /CapHeight, /Descent, /ItalicAngle, /StemV, /MissingWidth, /CharSet, /Rotate, /Resources, /Contents
Physical structure	%PDF, xref, startxref, trailer, /Size, /Prev, /Encrypt, /ID, %%EOF
Logical structure	/Root, /Catalog, /Pages, /Kids, /Parent, /Count, /Outlines, /Prev, /Next, /First, /Last
Content stream	stream, endstream, /Filter, /FlateDecode, /Length, /DecodeParms,
Metadata	/Info, /Author, /Producer, /Creator, /Keywords, /Title, /Subject, /ModDate, /CreationDate

## Appendix B

**Table A2:** Detailed Information on Extracted Basic Features.

Basic Feature Type	Feature Content
Objects (46)	num_obj, num_endobj, num_type, num_diff_type, maxnum_same_type, num_firstchar, num_lastchar, num_width, num_BaseFont, num_Subtype, num_Ascent, num_CapHeight, num_Descent, num_ItalicAngle, num_StemV, num_MissingWidth, num_CharSet, num_Rotate, num_Resources, num_Contents, loc_obj, oc_endobj, loc_firstchar, lloc_lastchar, loc_width, loc_BaseFont, loc_Subtype, loc_Ascent, loc_CapHeight, loc_CharSet, loc_Rotate, loc_Resources, loc_Contents, len_type, len_firstchar, len_lastchar, len_width, len_BaseFont, len_Subtype, len_Ascent, len_CapHeight, len_Descent, len_CharSet, len_Rotate, len_Resources, len_Contents
Physical structure (19)	version_pdf, num_xref, num_startxref, num_trailer, num_Size, num_Prev, num_Encrypt, num_ID, loc_xref, loc_startxref, loc_trailer, loc_Size, loc_Prev, loc_Encrypt, loc_ID, loc_EOF, len_xref, num_xref_modified, num_xref_initial
Logical structure (26)	num_Root, num_Catalog, num_Pages, num_Kids, num_Parent, num_Count, num_Outlines, num_Prev, num_Next, num_First, num_Last, loc_Root, loc_Catalog, loc_Pages, loc_Kids, loc_Parent, loc_Count, loc_Outlines, loc_Prev, loc_Next, loc_First, loc_Last, sum_Kids, sum_Parent, sum_Prev, sum_Next
Content stream (16)	num_stream, num_endstream, num_Filter, num_FlateDecode, num_Length, num_DecodeParms, len_stream, diff_num_Filter, diff_num_FlateDecode, diff_num_DecodeParms, loc_stream, loc_endstream, loc_Filter, loc_FlateDecode, loc_Length, loc_DecodeParms
Metadata feature (23)	len_Info, len_Author, len_Producer, len_Creator, len_Keywords, len_Title, len_Subject, int_ModDate, int_CreationDate, num_Info, num_Author, num_Producer, num_Creator, num_Keywords, num_Title, num_Subject, loc_Info, loc_Author, loc_Producer, loc_Creator, loc_Keywords, loc_Title, loc_Subject

## Appendix C

**Table A3:** Extracted malicious features.

Feature Type (Number)	Symbols
Basic attribute (26)	number of \x, +, space, character, string, getElementById, =, keyword, hex, int, encode, \, |, %, (), #, ., ’, [], {}, !, ;, length of shortest string, longest string, code line; proportion of keyword
Redirection (6)	number of referrer, setTimeout, replace, url, reload, location
Suspicious keyword (23)	number of exe, js, php, cmd, Wscript, string (length > 30), addEventListener, ActiveXObject, iframe, search, onbeforeunload, onbeforeload, setAttribute, fireEvent, dispatchEvent, onmouseover, onunload, onerror, classid, SystemRoot, attachEvent, createElement
Confusion (27)	number of charCodeAt, function, var, charAt, write, concat, fromCharCode, escape, eval, substring, indexOf, parseInt, toString, decode, random, log, split, heapspray, proportion of space, %, |, \, ;, (), hex, int, encode
